# Metabolomics of mammalian brain reveals regional differences

**DOI:** 10.1186/s12918-018-0644-0

**Published:** 2018-12-21

**Authors:** William T. Choi, Mehmet Tosun, Hyun-Hwan Jeong, Cemal Karakas, Fatih Semerci, Zhandong Liu, Mirjana Maletić-Savatić

**Affiliations:** 1 Program in Developmental Biology, Baylor College of Medicine, Houston, TX USA; 2 The National Library of Medicine Training Program in Biomedical Informatics, Houston, TX USA; 3 Medical Scientist Training Program, Baylor College of Medicine, Houston, TX USA; 4 Jan and Dan Duncan Neurological Research Institute, Texas Children’s Hospital, Houston, TX USA; 5 Department of Pediatrics-Neurology, Baylor College of Medicine, Houston, TX USA; 6 Department of Molecular and Human Genetics, Baylor College of Medicine, Houston, TX USA; 7 Quantitative Computational Biology Program, Baylor College of Medicine, Houston, TX USA; 8 Department of Neuroscience, Baylor College of Medicine, Houston, TX USA

**Keywords:** Metabolomics, Hippocampus, Frontal lobe, Cerebellum, Olfactory bulb, Network analysis

## Abstract

**Background:**

The mammalian brain is organized into regions with specific biological functions and properties. These regions have distinct transcriptomes, but little is known whether they may also differ in their metabolome. The metabolome, a collection of small molecules or metabolites, is at the intersection of the genetic background of a given cell or tissue and the environmental influences that affect it. Thus, the metabolome directly reflects information about the physiologic state of a biological system under a particular condition. The objective of this study was to investigate whether various brain regions have diverse metabolome profiles, similarly to their genetic diversity. The answer to this question would suggest that not only the genome but also the metabolome may contribute to the functional diversity of brain regions.

**Methods:**

We investigated the metabolome of four regions of the mouse brain that have very distinct functions: frontal cortex, hippocampus, cerebellum, and olfactory bulb. We utilized gas- and liquid- chromatography mass spectrometry platforms and identified 215 metabolites.

**Results:**

Principal component analysis, an unsupervised multivariate analysis, clustered each brain region based on its metabolome content, thus providing the unique metabolic profile of each region. A pathway-centric analysis indicated that olfactory bulb and cerebellum had most distinct metabolic profiles, while the cortical parenchyma and hippocampus were more similar in their metabolome content. Among the notable differences were distinct oxidative-anti-oxidative status and region-specific lipid profiles. Finally, a global metabolic connectivity analysis using the weighted correlation network analysis identified five hub metabolites that organized a unique metabolic network architecture within each examined brain region. These data indicate the diversity of global metabolome corresponding to specialized regional brain function and provide a new perspective on the underlying properties of brain regions.

**Conclusion:**

In summary, we observed many differences in the metabolome among the various brain regions investigated. All four brain regions in our study had a unique metabolic signature, but the metabolites came from all categories and were not pathway-centric.

**Electronic supplementary material:**

The online version of this article (10.1186/s12918-018-0644-0) contains supplementary material, which is available to authorized users.

## Background

Highly specialized brain functions, including learning, memory, attention and numerous other physiological processes, directly depend on the neuronal network formation, cellular homeostasis and overall tissue metabolism [1]. Metabolism is critical for the proper function of all living cells. The small biomolecules that participate in the metabolic processes determine an individual’s metabolic state and provide a close representation of that individual’s overall health status [2]. Recent studies have reported that the neurological and mental health disorders could be traced to alterations in the metabolic pathways [3, 4]. Pathologic conditions mostly disturb normal metabolic processes, resulting in changes that can be observed as metabolic signatures [5–12]. Tracing these metabolic signatures could thus reveal information about the physiologic state of the brain under a particular condition [13].

One of the approaches to trace metabolic signatures utilizes ‘omics methodologies, widely used for molecular profiling, identification of biomarkers, characterization of complex biochemical pathways, and examination of pathophysiological processes in various diseases [14]. One of the ‘omics sciences is metabolomics, which measures the biochemical content of cell processes downstream of genomic, transcriptomic, and proteomic systems [15–17]. The collection of all metabolites, known as the metabolome, includes a broad range of small (< 1 kDa) molecules such as monosaccharides, disaccharides and oligosaccharides; organic bases, nucleosides, and nucleotides; amino acids and peptides, numerous kinds of lipids, and other compounds [18]. The level of each metabolite within the metabolome depends on the specific physiological, developmental, and pathological state of a biological system, thus, reflecting on the phenotype in response to different genetic and environmental influences [19]. The systemic study of these small molecule metabolites thus may lead to a deeper insight into the dynamic phenotype of the biologic system and its change as a result of pathology [20, 21].

Mass spectrometry (MS) and nuclear magnetic resonance (NMR) spectrometry are the two technologies used for metabolomics studies [22]. MS can be combined with gas and liquid chromatography (GC and LC, respectively) separation tools to better resolve the metabolites [23]. Metabolomics analyses can generally be separated into two groups: targeted and untargeted analyses. Targeted metabolomics is used when a set of metabolites is examined, typically focusing on one or more selected pathways of interest [24]. Untargeted metabolomics involves simultaneously measuring as many metabolites as possible without bias [25]. In contrast to targeted metabolomics, untargeted metabolomics is global in scope and reveals the comprehensive metabolism of a whole cell/tissue/organism [26].

Despite the importance of the brain metabolism for its proper function and in pathology, our insights are sparse [27]. Thus, the objective of this study was to investigate whether various brain regions have diverse metabolome profiles, similarly to their genetic diversity. The answer to this question would suggest that not only the genome but also the metabolome may contribute to the functional diversity of brain regions. Further, abnormalities in the region-specific metabolome may be underlying the pathology, as recently reported [9]. We report here the complete metabolome profile of four mouse brain regions (olfactory bulb, frontal cortex, hippocampus, and cerebellum) involved in distinct brain functions (processing of smell, higher order functions, learning and memory, and movement, respectively), using an untargeted GC/LC-MS metabolomics analysis. We then sought to find the metabolites that distinguish each region using univariate, bivariate, and multivariate statistical approaches. We defined a set of metabolites that contribute to each region’s metabolic signature. To understand the metabolic architecture within each region, we concluded the study with a metabolic network analysis, identifying key modules with a potential to influence the metabolic network architecture.

## Methods

### Sample preparation

We harvested four different brain regions (olfactory bulb, frontal parenchymal, hippocampus, and cerebellum) from six 4-week-old C56BL6 mice. The tissue weight was measured and subsequently quickly frozen. Sample analysis was conducted by Metabolon, Inc. using a proprietary series of organic and aqueous extractions to remove the protein content while allowing maximum recovery of small molecules. The extract was divided into two parts: one for analysis by LC and the other for analysis by GC. TurboVap® (Zymark) was used to remove the organic solvent content. Each sample was then frozen and dried under vacuum. The following methodology section was provided by Metabolon, Inc. as their standard protocol for untargeted mass spectrometry metabolomics.

### Untargeted mass spectrometry profiling

Metabolon, Inc. used three independent platforms (ultrahigh performance liquid chromatography/tandem mass spectrometry (UHPLC/MS-MS^2^) optimized for basic species, UHPLC/MS-MS^2^ optimized for acidic species, and gas chromatography/mass spectrometry (GC/MS)) to generate untargeted high-throughput mass spectrometry-identified metabolites in the brain regions. Metabolic profiling analysis combined the three independent platforms using a non-targeted approach to obtain the relative quantity of a broad spectrum of molecules. Experimental samples and controls were randomized across platforms. In addition, several technical replicate samples were created from a homogeneous pool containing a small amount of all study samples. Prior to extraction, recovery standards were added to ensure quality control (QC) charts. Sample preparation was conducted using a Metabolon, Inc. proprietary series of organic and aqueous extractions to remove the protein content while allowing maximum recovery of small molecules. Each sample was then frozen and dried under vacuum. A number of additional samples were included with each day’s analysis for QA/QC charts. Furthermore, a selection of QC compound was added to each sample, including those under test. These compounds were cautiously chosen so as not to interfere with the measurement of the endogenous compounds. Prior to loading the samples into the mass spectrometers, the instrument variability was determined by calculating the median relative standard deviation (RSD) for the standards that were added to all sample. Overall variability was determined by calculating the median RSD for all endogenous metabolites (i.e., non-instrument standards) present in 100% of the samples, which are technical replicates of pooled samples. For UHPLC/MS/MS^2^ analysis, aliquots were separated using a Waters Acquity UPLC (Waters Corp.) and analyzed using an LTQ mass spectrometer (MS) (Thermo Fisher Scientific, Inc.), which consisted of an electrospray ionization source and linear ion-trap mass analyzer. The MS instrument scanned 99 to 1,000 m/z and alternated between MS and MS^2^ scans using dynamic exclusion with approximately 6 scans per second. Derivatized samples for GC/MS were loaded to a 5% phenyldimethyl silicone column with helium as the carrier gas and a temperature ramp from 60 °C to 340 °C and then analyzed on a Thermo-Finnigan Trace DSQ MS (Thermo Fisher Scientific, Inc.) operated at unit mass resolving power with electron impact ionization and a 50 to 750 amu scan range.

### Metabolite identification

Metabolites were identified by comparison of the ion features in the experimental samples with a library of compound standard entries that included retention time, molecular weight to charge ratio (m/z), preferred adducts, and in-source fragments as well as associated MS spectra, and were curated by visual inspection for quality control using the software improved by Metabolon, Inc. [28]. The raw mass spectrometry data extraction gave information that could be loaded into a relational database. Afterward, the information was examined, and appropriate QC limits were implemented. Numerous curation procedures were carried out to ensure that a high-quality dataset was made available for statistical analysis and data interpretation. QC and curation processes were generated to ensure precise and consistent identification of true compound entities, and to remove those representing system artifacts, mis-assignments, and background noise. Metabolon, Inc. uses proprietary visualization and interpretation software to confirm the consistency of peak identification among the various samples. Library matches for all compounds were checked for each sample and corrected if necessary.

### Weighted correlation network analysis

To better understand the metabolite network organization in the brain, we performed the weighted correlation network analysis (WGCNA). This method is a network inference algorithm derived from a biological profile and widely applied for studying biological networks [29]. This algorithm relies on the pairwise correlation between metabolites and it provides information such as network module (a subset of metabolites that highly correlate each other) and eigen-metabolite (an imaginary metabolite that represents a module). To perform the network analysis, we first calculated every pairwise correlation of metabolites from the metabolite profiles of the entire 24 samples of four brain regions in this study. All of the correlations were stored in the matrix *S*, and *s*_*ij*_ stored a correlation between i-th metabolite and j-th metabolite in the profile. Next, we defined a weighted network adjacency *A* with the soft thresholding manner [29]. An element of i-th and j-th metabolites in soft-thresholded adjacency weighted matrix *A* is defined by ${a}_{ij}={s}_{ij}^{\beta }$. *β* ≥ 1 is a parameter to fit the network of *A* to the scale-free topology, which many biological networks follow [30]. For a scale-free network, degree/connectivity distribution of the network follows a power low *p*(*k*)~*k*^−*γ*^, where *p*(*k*) is the distribution of nodes with a degree of *k* in the network. From an empirical observation of the scale-free topology with different *β* values, we chose 11 as the optimal value of the *β*. We next defined Ω, which reflects a relative inter-connectedness between a pair of metabolites:

$$ {\omega}_{ij}=\frac{l_{ij}+{a}_{ij}}{\min \left({k}_i,{k}_j\right)+1-{a}_{ij}} $$

where ${l}_{ij}={\sum}_u{a}_{iu}\ast {a}_{uj}$ and the node connectivity ${k}_i={\sum}_u{a}_{ij}$.

Ω is converted to a dissimilarity matrix *D*, and *D* is defined by *d*_*ij*_ = 1 − *ω*_*ij*_.

With the dissimilarity matrix we performed a complete-linkage hierarchical clustering method using ‘flashClust’ function in R [31], and then we cut the hierarchical tree using the dynamic branch cut method [32] using ‘cutDynamic’ function in R and default parameters of the function. After the tree-cutting, we defined a metabolite module if two criteria were satisfied: i) metabolites in the module were connected in the cut tree, and ii) the number of metabolites in the module was larger than 10. If metabolites were not assigned to any module, they were omitted. We repeated the clustering and cutting procedure until every metabolite was assigned. At the final step, we calculated eigen-metabolite for each network module, defined as the first principal component of the concentration matrix of the metabolites in the module.

### Bioinformatics and statistical analyses

Statistical analysis was conducted using ‘R’ language (http://cran.r-project.org/). Analysis of variance (ANOVA) with false detection rate (FDR) correction using Benjamini–Hochberg procedure was performed for the metabolomics data [33, 34] Normalization when indicated was done using studentized residual or z-score. Comparison of the statistical difference in a single metabolite between two regions, we used Welch’s two-sample t-test.

## Results

### Brain metabolome is enriched in several classes of metabolites

**Fig. 1:**
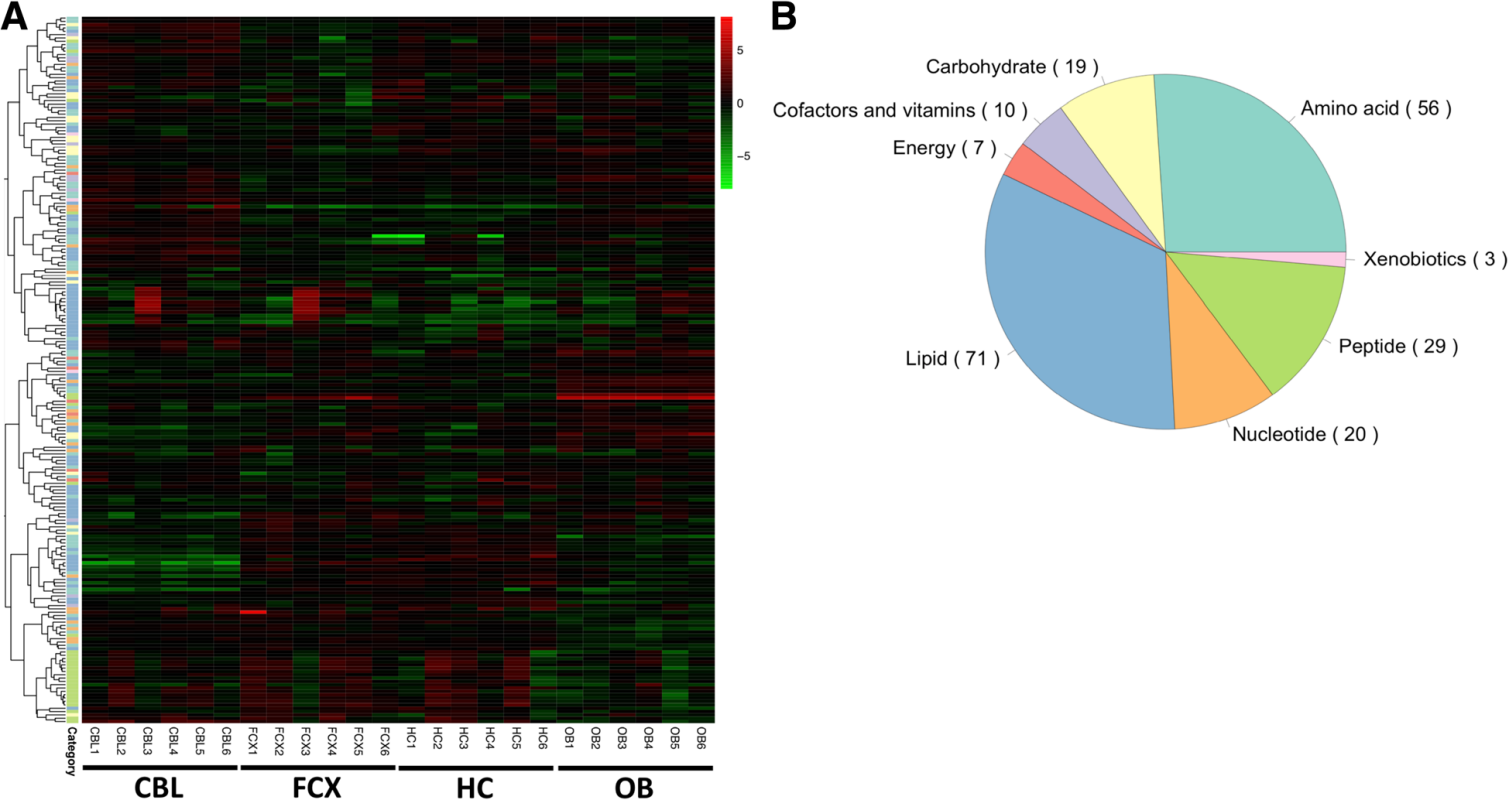
Untargeted metabolomics identifies 215 metabolites in four brain regions of an adult mouse. From a proprietary library of Metabolon, Inc. containing > 2000 compounds, 215 metabolites were identified in the four brain regions: olfactory bulb, frontal cortex, hippocampus and cerebellum (*N* = 6 per region). **a.** Heat map represents the relative concentration of each metabolite organized by its respective metabolic category and brain region. Relative concentration of the metabolites was normalized by using studentized residual method. Color gradient represents the Z score distribution of each metabolite across all four regions, each containing six biological samples. Within-group variance is marginal. **b.** Eight major types of metabolites were identified. Shaded regions indicate their respective metabolic category and the number of metabolites in shown in parentheses. OB, olfactory bulb; FCX, frontal cortex; HC, hippocampus; CB, cerebellum

Using UHPLC/MS-MS^2^ optimized for basic species, UHPLC/MS-MS^2^ optimized for acidic species, and GC/MS, we performed untargeted high-throughput mass spectrometry and identified metabolites in four mouse brain regions: olfactory bulb, frontal cortex, hippocampus, and cerebellum (*N* = 6 per region). We detected 215 metabolites overall (Fig. 1)*,* using a library of more than 2000 purified compounds. Mean concentration-variance plot indicated marginal within-group variance. The 215 metabolites belonged to eight different categories of small molecules: amino acids, carbohydrates, cofactors and vitamins, energy metabolism, lipids, nucleotides, peptides, and xenobiotics. Amino acid and lipid categories predominated (Fig. 1b).

To then determine the metabolites that differed significantly across all regions, we used analysis of variance (ANOVA) and false discovery rate (FDR) for multiple testing corrections at a cutoff of FDR < 0.01. Seventy metabolites achieved this statistical significance**.** We then sought to determine whether the degree of abundance in these 70 significant metabolites could be used to infer region specificity (Additional file 1)**.** We used a two-sample t-test to examine pairwise difference in 70 metabolites for each brain region (the threshold *p*-value < 0.05 was used as a threshold for statistical significance, and log2 fold change of metabolite concentration between any two regions was used to calculate the relative metabolite abundance). 27/70 metabolites were detected in either high (log2 fold-change > 0) or low (log2 fold-change < 0) amounts in the cerebellum, and 9/70 metabolites were highly abundant in the olfactory bulb. In the hippocampus, two metabolites were either high or low, and in the frontal cortex no metabolites were significantly different. Although cerebellum, olfactory bulb and hippocampus (to an extent) may have a set of metabolites that can distinguish them from each other, the abundance of each metabolite was not a sufficient analytical parameter to distinguish the various brain regions. Regardless, a region-specific metabolome relationship still appeared to exist.

#### Untargeted mass spectrometry suggest regional metabolic differences

**Fig. 2:**
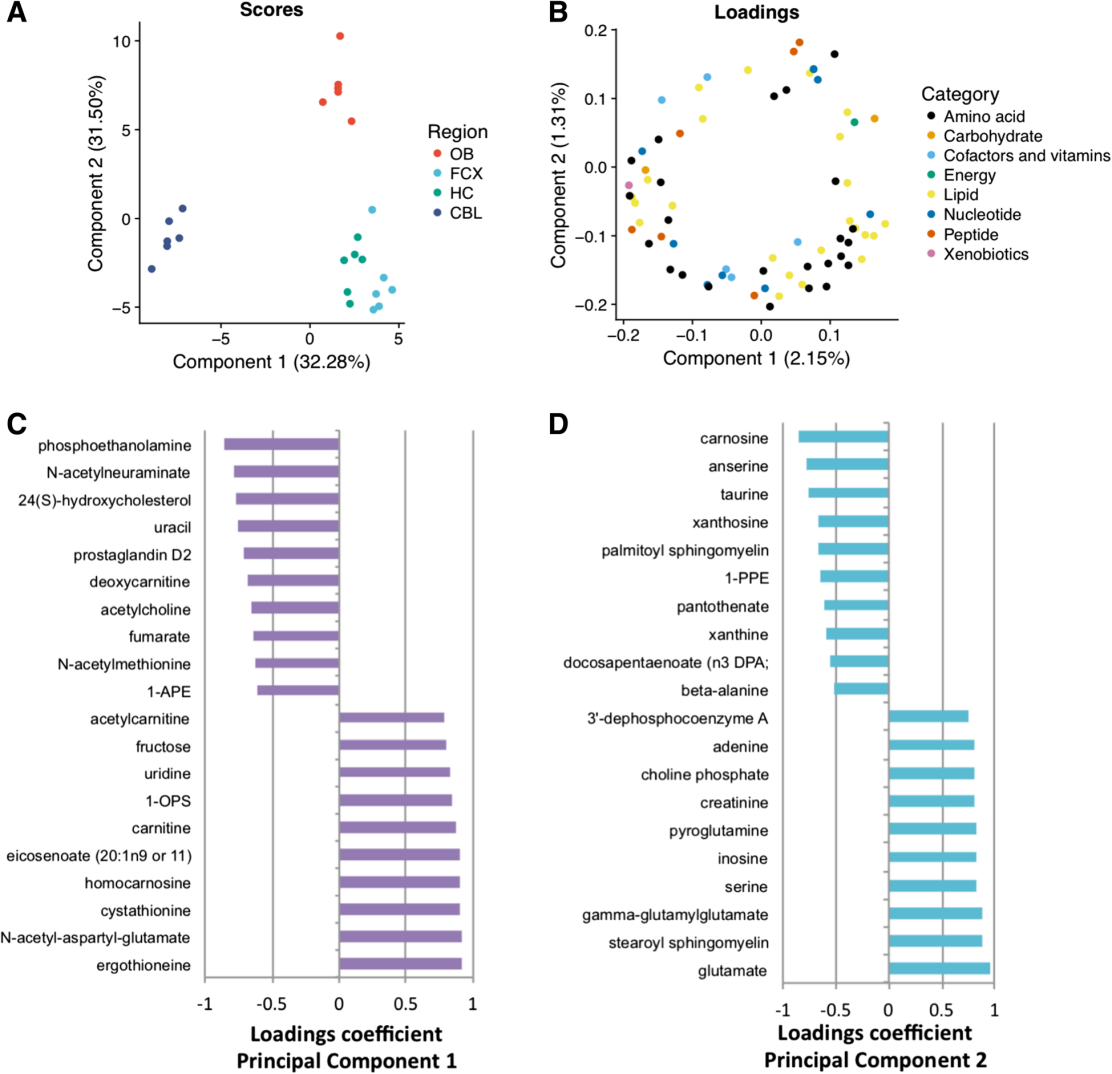
Multivariate analysis via PCA reduces the complexity of the brain region metabolome and identifies key components that contribute to their differences. **a.** Scattered scores plot shows the degree of separation across all brain region samples when metabolites with real significance are analyzed. **b.** Scattered loadings plot highlights the category of metabolites that contribute to the separation of the brain region clusters. **c-d.** The top 10 metabolites that explain the metabolome differences of the brain regions based on principal components 1 and 2. OB, olfactory bulb; FCX, frontal cortex; HC, hippocampus; CBL, cerebellum

To further parse out the metabolic profiles of four brain regions, we applied a multivariate analysis utilizing PCA **(**Fig. 2**)**. With the set of metabolomics data, PCA grouped the brain regions into a few latent components. These components identified the brain regions with strong similarity; therefore, brain samples with strong similarity would share the same component, while those that are different would be separated by a distance. As seen in Fig. 2a, the separation of the brain regions is evident in the first and second principal components. While the olfactory bulb and cerebellum were distinctly clustered in the scores plot, hippocampus and the frontal cortex clustered together, suggesting that they share similar metabolome. Based on the loadings, we revealed a set of metabolic profiles for each brain region **(**Fig. 2b-d; Additional file 2).

### Targeted analysis of the brain Metabolome

**Fig. 3:**
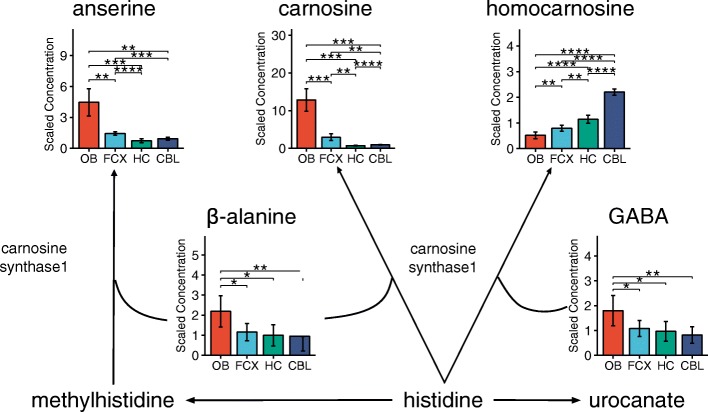
Histidine-containing dipeptides, except homocarnosine, are enriched in olfactory bulb. Homocarnosine is highly enriched in the cerebellum. Other regions do not have significant amounts of these dipeptides. Bar graphs are mean ± SD metabolite concentration. Asterisks (*) indicates statistical significance between the regions estimated by Welch’s two-sample t-test. (*: *p* ≤ 0.05, **: *p* ≤ 0.01, ***: *p* ≤ 0.001, ****: *p* ≤ 0.0001). OB, olfactory bulb; FCX, frontal cortex; HC, hippocampus; CBL, cerebellum

**Fig. 4:**
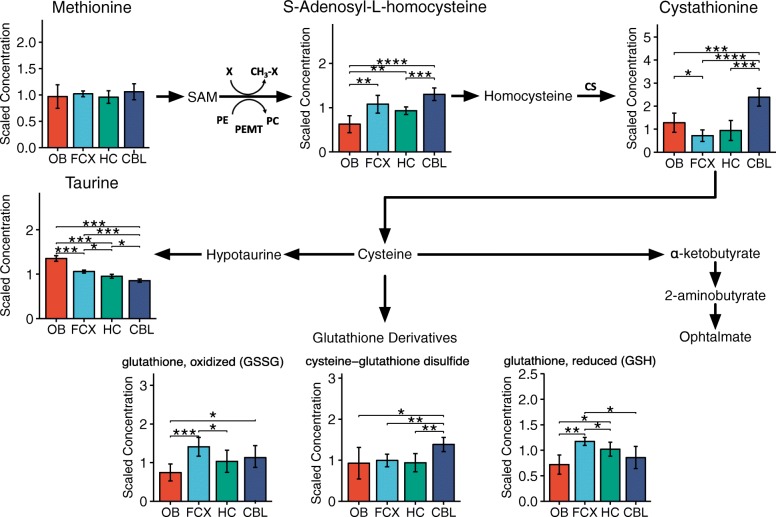
Cysteine pathway metabolites are enriched in cerebellum. Bar graphs are mean±SD metabolite concentration. Asterisks (*) indicates statistical significance between the regions estimated by Welch’s two-sample t-test. (*: *p* ≤ 0.05, **: *p* ≤ 0.01, ***: *p* ≤ 0.001, ****: *p* ≤ 0.0001). OB, olfactory bulb; FCX, frontal cortex; HC, hippocampus; CBL, cerebellum. SAM: S-Adenosyl methionine; PE: Phosphatidylethanolamine; PC: Phosphatidylcholine; PEMT: PE N-methyltransferase; CS: Cystathionine-β-synthase

We then focused on individual metabolites among those significantly different between brain regions, to examine whether they belong to biochemically defined pathways. We found significantly high levels of histidine-containing dipeptides, carnosine and anserine, in the olfactory bulb relative to the other regions of the brain (Fig. 3**;** *p* < 0.001). It has been proposed that carnosine plays a role as a neuromodulator in olfaction [35], and our findings support this hypothesis given the abundance of this metabolite in this region compared to others. Further, we found that metabolites that participate in cysteine pathway were differently distributed in different regions (Fig. 4). Cysteine is a non-essential amino acid central to many biochemical pathways including the biosynthesis of antioxidants glutathione and taurine and production Coenzyme A (CoA) [36]. Cystathionine, an intermediate product of cysteine, was present in significantly higher amount in the cerebellum than in the other regions (*p* < 0.01), as reported [37]. In addition, oxidized glutathione level was the highest in the frontal region (*p* < 0.05-p < 0.001), while cysteine-glutathione disulphide was higher in cerebellum compared to other regions (p < 0.01). These data suggest that cerebellum may experience relatively elevated oxidative stress and anti-oxidant demands matched by increased activity along the cysteine transulfuration pathway to generate glutathione. Cysteine and glutathione have beneficiary effects on nerve cell survival by reducing the oxidative stress [38–41] and have been reported as some of the key metabolites in Parkinson’s and Huntington’s diseases [27]. Taurine, also derived from cysteine, has been implicated in multiple cellular functions in the brain including a central role as a neurotransmitter, neuromodulator, an osmolyte, and as a neuroprotectant against oxidative stress. The highest level of taurine was observed in the olfactory bulb followed by the frontal cortex, and the lowest level was observed in the cerebellum. These region-specific taurine levels suggest differential importance for this neuroactive amino acid derivative across the brain regions analyzed.

**Fig. 5:**
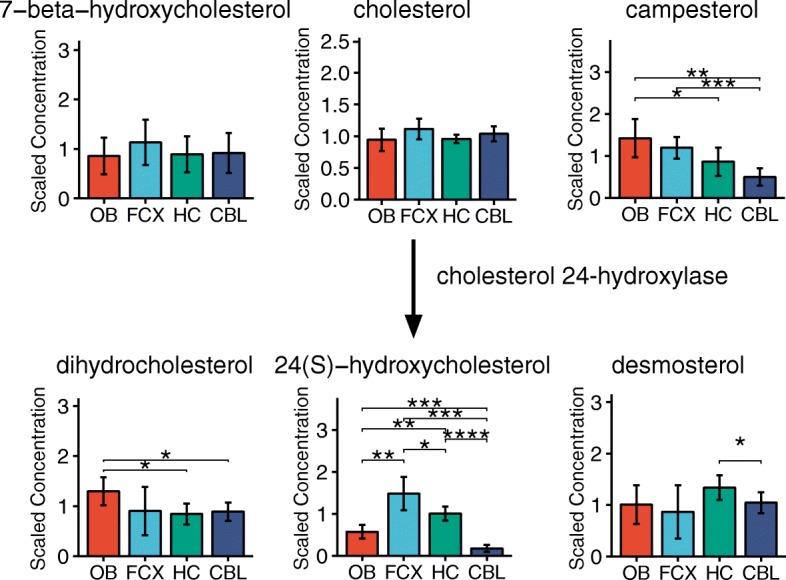
While cholesterol is evenly distributed in all brain regions studied, it is converted to 24S-cholesterol mostly in the cortical regions and hippocampus. Two plant-based cholesterol derivatives, campesterol and desmosterol, are also detected. Bar graphs are mean ± SD metabolite concentration. Asterisks (*) indicates statistical significance between the regions estimated by Welch’s two-sample t-test. (*: *p* ≤ 0.05, **: *p* ≤ 0.01, ***: *p* ≤ 0.001, ****: *p* ≤ 0.0001). OB, olfactory bulb; FCX, frontal cortex; HC, hippocampus; CBL, cerebellum

Interestingly, the levels of cholesterol in the brain regions examined did not vary **(**Fig. 5**).** Cholesterol in the brain is synthesized de novo and its concentration is regulated by the rate of its turnover. Generation of 24-S-cholesterol by cholesterol-24-dehydrogenase enzyme in the endoplasmic reticulum is the main pathway for cholesterol turnover in the brain [42]. 24-S-hydroxycholesterol crosses the blood brain barrier and is transported via circulation to the liver for further metabolism [43]. In our study, the highest level of 24-S-cholesterol was found in the frontal cortex followed by the hippocampus (*p* < 0.01), implying a higher rate of cholesterol metabolism in these tissues relative to the cerebellum and the olfactory bulb (Fig. 5). Dietary cholesterol homologues from plants, campesterol and sitosterol, are known to get enriched to some extent in the mammalian brain [44] and were detected in our samples as well. The campesterol was most abundant in the olfactory bulb (Fig. 5; *p* < 0.001). Brain cholesterol metabolism seems to play a role in the Alzheimer’s disease pathogenesis. It was shown that both beta-amyloid and amyloid precursor protein can oxidize cholesterol to form 7-beta-hydroxycholesterol, a proapoptotic oxysterol that is neurotoxic at nanomolar concentrations [45]. Our data indicate that this metabolite is produced in the normal brain under physiological conditions and it will be important to study this metabolite in mouse models of neurodegeneration.

**Fig. 6:**
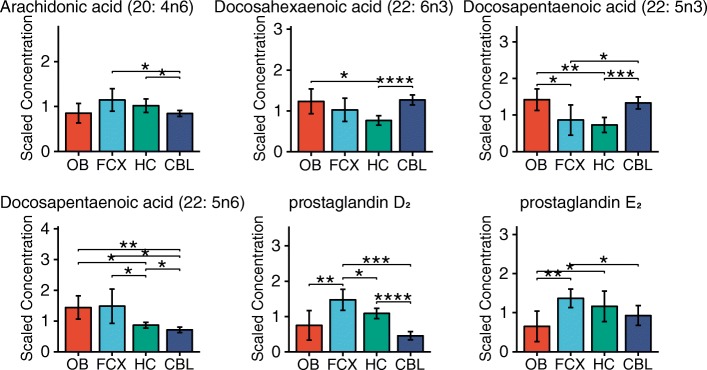
The distribution of poly-unsaturated fatty acids and prostaglandins in different brain regions. Bar graphs are mean ± SD metabolite concentration. Asterisks (*) indicates statistical significance between the regions estimated by Welch’s two-sample t-test. (*: *p* ≤ 0.05, **: *p* ≤ 0.01, ***: *p* ≤ 0.001, ****: *p* ≤ 0.0001). OB, olfactory bulb; FCX, frontal cortex; HC, hippocampus; CBL, cerebellum

Finally, the brain tissue was particularly rich in two polyunsaturated fatty acids (PUFAs), arachidonic acid (AA) (20:4n-6) and docosahexaenoic acid (DHA) (22:6n-3) **(**Fig. 6**)**. The DHA and AA are essential for brain function, optimal growth, and development [46]. PUFAs are also very effective against post-stroke brain injury and angiogenesis and they support white matter restoration [47]. The differences in PUFA distribution between the cerebellum and the other regions of the brain may come from the composition of cellular membranes of Purkinje cells and granule cells found in the cerebellum. The high levels PUFAs in Purkinje cells provide protection against the degeneration and autophagy at some pathologic conditions [48]. Joffre et al. found highest concentration of DHA and AA in the hypothalamus in their mouse model study [49]. It has also been reported that in the mouse astrocytes, prostaglandin D2 and prostaglandin E2 are powerful inducers of nerve growth factor and brain derived neurotrophic factor [50].

### Global metabolic connectivity and network module analysis of the brain Metabolome

**Fig. 7:**
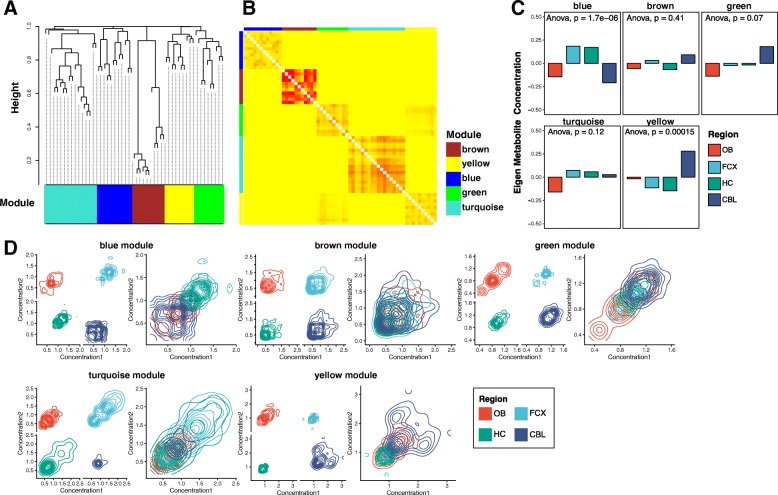
Metabolite correlation classifies the metabolome into brain region-specific network modules. **a.** The weighted correlation network analysis (WGCNA) cluster dendrogram identifies 61 out of 215 metabolites across four different brain regions as five distinct network modules. **b.** The heatmap of the topological overlap matrix of the metabolite networks shows that the intra-cluster similarities of each module are high, confirming the clustering in A. **c.** Eigen-metabolites of each module exhibit different concentration patterns. The *p*-values were calculated by ANOVA. **d.** The contour graphs of correlations of every pairwise metabolite in each module shows the correlations in each brain region as well as in all of them merged together (rightmost graph for each module). While there is a high correlation overall (rightmost graph for each module), the decomposed graphs show a unique correlation pattern for each brain region. The divergent patterns of any given module are also different from patterns of every other module

To examine whether each brain region has a distinct metabolic architecture in addition to its enrichment in certain metabolites, we performed WGCNA analysis on all 215 metabolites. We found five modules consisting of the 61 out of 215 metabolites **(**Fig. 7a**;** Additional file 3). 154 metabolites had low correlation to other metabolites and were thus omitted from network analyses**.** The correlation of all pairwise metabolites in each module is high **(**Fig. 7b**).** Although we used WGCNA to detect the network with high correlation across the entire dataset, we discovered that each module in the network has brain region-specific property. Namely, we calculate eigen-metabolites of each module using principal component analysis of metabolite concentrations within each module **(**Fig. 7c**).** The eigen-metabolites show that each module has different pattern of metabolite concentration compared to other modules, and these patterns differed for each brain region. Region-specific differences in two modules, the blue and yellow ones, was confirmed with ANOVA. Further, the eigen-metabolites identified hub metabolites for each module with the $k$ connectivity measure: deoxy-carnitine (blue, k = 0.954), 2-palmitoyl glycerol phosphoethanolamine (brown, k = 0.999), glycine (green, k = 0.968), leucil-leucine (turquoise, k = 0.968), and ergothioneine (yellow, k = 0.942). To then examine how each brain region builds its metabolic architecture around these hub metabolites, we plotted the correlations of every pairwise metabolite of each module in the contour graph **(**Fig. 7d**)**. Indeed, each module except the brown one shows different correlation pattern for each brain region. These data indicate that not only brain regions differ in a set of metabolites they accumulate as we have shown using a traditional, targeted approach, but also in their organization of metabolic networks centered around a few common hub metabolites as shown using the WGCNA analysis.

## Discussion

In this study, we utilized several approaches to examine brain metabolome. We investigated whether different brain regions have unique metabolome contents using untargeted mass spectrometry metabolomic profiling of the mouse brain. First, we found that each region is enriched in a set of metabolites, supporting our hypothesis that metabolic specificity may be important for the biological function of a given region. Not surprisingly, the biochemical profiles of the frontal and hippocampal regions were very similar, while the cerebellum was the most distinct when compared to other tissues. Second, we found that each region has a unique metabolic network architecture, further highlighting their metabolic specificity.

Metabolomics has become one of the approaches to understand the integrated response of cellular processes to genetic and environmental factors. However, given the large amount of information generated through metabolomics, as in other ‘omics approaches, the study of metabolism requires additional approaches to reduce complexities. Namely, understanding the metabolome can be a daunting task because of the hundreds of measured metabolite species. A classical way to study metabolism is *pathway-centric*. The biochemical pathways provide the roadmap for energy transfer, which includes the enzymes catalyzing a reaction, the substrates that get converted from one state to another, and the physical chemistry, i.e. kinetics and thermodynamics, that explains why and how the energy transfer can take place. In our study, many biochemical differences were observed among the various brain regions, illustrating the diversity of global metabolism corresponding to specialized regional brain function. Overall, olfactory bulb and cerebellum showed more distinct metabolic profiles, while the cortical parenchymal and hippocampus were more similar. This is not surprising, given that both structures participate in memory formation. Among the notable differences across all four regions were distinct redox status and region-specific fatty acid profiles, suggesting that different brain regions depend on these molecules to a varying degree to perform their function and maintain steady-state.

When one moves away from the pathway-centric approach and starts to incorporate the interconnected metabolic pathways, the complexities increase exponentially. For example, the metabolite concentrations are theoretically determined by the activity of the enzymes. However, there are countless variables that both the enzyme activity and the metabolites are affected by. Understanding the quantity of just one metabolite and/or its interaction with a few other metabolites that belong to the same pathway is not enough to understand metabolism of a given cell or tissue. On the other hand, by looking at the whole metabolome, we can find characteristic patterns in metabolite profiles, directly linking them to the underlying biochemical reaction *networks*. We thus reasoned, based on the biochemistry paradigm of feedback regulation, that the metabolites could be part of a biochemical network of interconnecting pathways where the changes of a set of metabolites could influence another set of metabolites. In theory, the metabolites in a biochemical network are connected with each other; a change in one metabolite can influence a metabolite from a different pathway, thus creating a dense network. With the relative concentrations of the identified metabolites, a partial relationship could be inferred by the correlations between metabolites [51, 52]. The weighted correlation network analysis (WGCNA) [29] we used to identify network modules of the metabolites showed exactly what we predicted – each brain region has a unique metabolic network architecture.

## Conclusion

We observed many differences in the metabolome among the various brain regions investigated. All four brain regions in our study had a unique metabolic signature, but the metabolites came from all categories and were not pathway-centric. To better understand these unique, region-specific metabolic signatures, the metabolic network analysis essentially found network of structures, and led to the discovery of the five hubs important to maintain the common metabolic architecture for all brain regions.

## Additional files

Additional file 1: 70 metabolites significantly differed across all brain regions. The significance of the metabolites was determined by ANOVA with FDR correction (FDR < 0.01). (PDF 2060 kb)

Additional file 2: List of predominant metabolites of the four brain regions determined by PCA loadings. (PDF 52 kb)

Additional file 3: Metabolites that represent each identified module. (PDF 50 kb)

## Abbreviations

AA: Arachidonic acid
ANOVA: Analysis of variance
CoA: Coenzyme A
DHA: Docosahexaenoic acid
DSQ: Dual stage quadrupole
FDR: False detection rate
GC: Gas chromatography
kDa: Kilodalton
LC: Liquid chromatography
LTQ: Linear Trap Quadropole
m/z: Molecular weight to charge ratio
MS: Mass spectrometry
NMR: Nuclear magnetic resonance
PCA: Principal component analysis
PUFAs: Polyunsaturated fatty acids
QA/QC: Quality assessment/Quality control
RSD: Relative standard deviation
UHPLC/MS-MS^2^: Ultrahigh performance liquid chromatography/tandem mass spectrometry
WGCNA: Weighted correlation network analysis
