# Supplementary material for: Metabolomics of mammalian brain reveals regional differences
Source: BMC Syst Biol. 2018 Dec 21;12(Suppl 8):127. doi: 10.1186/s12918-018-0644-0 (PMC6302375; doi:10.1186/s12918-018-0644-0)
Supplement: Supplementary file 1 — 70 metabolites significantly differed across all brain regions. The significance of the metabolites was determined by ANOVA with FDR correction (FDR < 0.01). (PDF 2060 kb) [file 12918_2018_644_MOESM1_ESM.pdf]

Rank	metabolite	p-value	FDR
1	homocarnosine	9.65E-15	2.06E-12
2	taurine	2.15E-13	2.30E-11
3	carnosine	2.28E-11	1.63E-09
4	N-acetylneuraminate	1.26E-10	6.76E-09
5	ergothioneine	5.73E-09	2.45E-07
6	anserine	1.38E-08	4.22E-07
6	phosphoethanolamine	1.29E-08	4.22E-07
8	eicosenoate (20:1n9 or 11)	2.54E-08	6.78E-07
9	N-acetyl-aspartyl-glutamate (NAAG)	2.93E-08	6.96E-07
10	24(S)-hydroxycholesterol	4.33E-08	8.64E-07
10	glutamate	4.44E-08	8.64E-07
12	deoxycarnitine	1.10E-07	1.96E-06
13	1-oleoylglycerophosphoserine	3.79E-07	6.24E-06
14	pyroglutamine*	4.65E-07	7.11E-06
15	carnitine	9.49E-07	1.35E-05
16	cystathionine	1.08E-06	1.44E-05
17	3-(4-hydroxyphenyl)lactate	2.00E-06	2.26E-05
17	hydroxyisovaleroyl carnitine	1.91E-06	2.26E-05
17	uridine	1.86E-06	2.26E-05
20	creatine	3.53E-06	3.78E-05
21	phosphopantetheine	4.08E-06	4.15E-05
22	S-adenosylhomocysteine (SAH)	5.27E-06	4.97E-05
22	xanthine	5.35E-06	4.97E-05
24	adenine	5.99E-06	5.34E-05
25	xanthosine	6.40E-06	5.48E-05
26	creatinine	9.05E-06	7.45E-05
27	gamma-glutamylglutamate	1.83E-05	1.41E-04
27	prostaglandin D2	1.85E-05	1.41E-04
29	pantothenate	2.04E-05	1.50E-04
30	acetylcarnitine	2.15E-05	1.54E-04
31	2-hydroxyglutarate	2.23E-05	1.54E-04
32	1-palmitoylplasmenylethanolamine*	2.77E-05	1.85E-04
33	3'-dephosphocoenzyme A	2.96E-05	1.92E-04
34	choline phosphate	3.21E-05	2.02E-04
35	allo-threonine	4.22E-05	2.58E-04
36	palmitoyl sphingomyelin	5.06E-05	3.01E-04
37	fructose	6.02E-05	3.48E-04
38	stearoyl sphingomyelin	8.05E-05	4.53E-04
39	coenzyme A	8.61E-05	4.73E-04
40	acetylcholine	1.06E-04	5.36E-04
40	N-acetylmethionine	1.02E-04	5.36E-04
40	propionylcarnitine	1.08E-04	5.36E-04
40	uracil	1.08E-04	5.36E-04
44	2-aminoadipate	1.15E-04	5.57E-04
45	threonylleucine	1.45E-04	6.88E-04
46	fumarate	3.40E-04	1.58E-03
47	N-acetylaspartate (NAA)	4.10E-04	1.86E-03
47	serine	4.17E-04	1.86E-03
49	campesterol	5.47E-04	2.39E-03
50	1-arachidonoylglycerophosphoinositol*	5.85E-04	2.51E-03
51	1-arachidonoylglycerophosphoethanolamine*	7.37E-04	2.88E-03
51	5-hydroxyindoleacetate	7.79E-04	2.88E-03
51	5-methylthioadenosine (MTA)	7.21E-04	2.88E-03
51	aspartate	7.77E-04	2.88E-03
51	citrulline	7.26E-04	2.88E-03
51	docosapentaenoate (n3 DPA; 22:5n3)	7.56E-04	2.88E-03
51	glutathione, reduced (GSH)	6.91E-04	2.88E-03
51	guanosine	7.75E-04	2.88E-03
59	inosine	7.95E-04	2.89E-03
60	gamma-glutamylglutamine	8.58E-04	3.06E-03
61	docosapentaenoate (n6 DPA; 22:5n6)	1.16E-03	4.08E-03
62	alanine	1.44E-03	4.98E-03
63	1,2-dipalmitoylglycerol	1.58E-03	5.33E-03
63	nicotinamide adenine dinucleotide (NAD+)	1.59E-03	5.33E-03
65	methylphosphate	1.63E-03	5.37E-03
66	glutaroyl carnitine	1.99E-03	6.47E-03
67	beta-alanine	2.30E-03	7.34E-03
68	glutathione, oxidized (GSSG)	2.42E-03	7.61E-03
69	oleate (18:1n9)	2.92E-03	9.07E-03
70	docosahexaenoate (DHA; 22:6n3)	3.07E-03	9.37E-03
