# Supplementary material for: Metabolomics of mammalian brain reveals regional differences
Source: BMC Syst Biol. 2018 Dec 21;12(Suppl 8):127. doi: 10.1186/s12918-018-0644-0 (PMC6302375; doi:10.1186/s12918-018-0644-0)
Supplement: Supplementary file 2 — List of predominant metabolites of the four brain regions determined by PCA loadings. (PDF 52 kb) [file 12918_2018_644_MOESM2_ESM.pdf]

Olfactory Bulb	Frontal Parenchyma	Hippocampus	Cerebellum
-Campesterol	-N-acetylaspartate (NAA)	-5-hydroxyindoleacetate	-Citrulline
-Taurine	-1,2-dipalmitoylglycerol	-Nicotinamide adenine dinucleotide (NAD+)	-Uridine
-Xanthine	-1-arachidonoylglycero-phosphoethanolamine	-2-hydroxyglutarate	-Cystathionine
-Xanthosine	-Acetylcholine	-1-arachidonoylglycerophosphoinositol	-Fructose
-1-palmitoylplasmenyl-ethanolamine	-N-acetylmethionine	-Glutaroyl carnitine	-Acetylcarnitine
-Carnosine	-5-hydroxyindoleacetate	-Glutathione, reduced (GSH)	-2-aminoadipate
-Anserine	-Nicotinamide adenine dinucleotide (NAD+)	-Coenzyme A	-Ergothioneine
-beta-alanine	-allo-threonine	-Glutathione, oxidized (GSSG)	-N-acetyl-aspartyl-glutamate (NAAG)
-5-methylthioadenosine (MTA)	-2-hydroxyglutarate	-Guanosine	-Eicosenoate (20:1n9 or 11)
-Palmitoyl sphingomyelin	-Alanine	-Propionylcarnitine	-Carnitine
