# Supplementary material for: Metabolomics of mammalian brain reveals regional differences
Source: BMC Syst Biol. 2018 Dec 21;12(Suppl 8):127. doi: 10.1186/s12918-018-0644-0 (PMC6302375; doi:10.1186/s12918-018-0644-0)
Supplement: Supplementary file 3 — Metabolites that represent each identified module. (PDF 50 kb) [file 12918_2018_644_MOESM3_ESM.pdf]

| Blue Module | Brown Module | Green Module | Turquoise Module | Yellow Module |
|---|---|---|---|---|
| -Phosphoethanolamine<br>-24(S)-hydroxycholesterol<br>-Deoxycarnitine<br>-Pyroglutamine<br>-3-(4-hydroxyphenyl) lactate<br>-Choline phosphate<br>-Acetylcholine<br>-N-acetylmethionine<br>-Uracil<br>-Serine<br>-Alanine<br>-Glutaroyl carnitine | -2-docosapentaenoyl glycerol phosphoethanolamine<br>-2-oleoyl glycerol phosphocholine sphingosine<br>-1-stearoyl glycerol phosphocholine<br>-1-oleoyl glycerol phosphocholine<br>-2-palmitoyl glycerol phosphocholine<br>-1-palmitoyl glycerol phosphocholine<br>-2-arachidonoyl glycerol phosphoethanolamine<br>-2-arachidonoyl glycerol phosphocholine<br>-2-palmitoyl glycerop hosphoethanolamine<br>-2-docosahexaenoyl glycerol phosphoethanolamine | -Cysteine<br>-Cysteine-glutathione disulfide<br>-Phenylalanine<br>-Tyrosine<br>-Glycine<br>-N1-methyladenosinea<br>-Glutamine<br>-Cytidine 5'-monophosphate (5'-CMP)<br>-Nicotinamide<br>-5-oxoproline | -Isoleucylleucine<br>-Leucylglycine<br>-Isoleucylserine<br>-Leucylserine<br>-Leu-leu-leu<br>-Leucylasparagine<br>-Phenylalanylleucine<br>-Leucylleucine<br>-Glycylleucine<br>-Leucylglutamate<br>-Aspartylleucine<br>-Isoleucylglutamate<br>-Leucylalanine<br>-Alanylleucine<br>-Serylleucine<br>-Threonylphenylalanine<br>-Serylphenyalanine<br>-Valylphenylalanine | -Ergothioneine<br>-Cystathionine<br>-Uridine<br>-Fructose<br>-2-aminoadipate<br>-Citrulline<br>-Threonine<br>-N-acetylglutamine<br>-Tryptophan<br>-Cystine |
